# Shared Active Site Architecture between the Large Subunit of Eukaryotic Primase and DNA Photolyase

**DOI:** 10.1371/journal.pone.0010083

**Published:** 2010-04-09

**Authors:** Ludovic Sauguet, Sebastian Klinge, Rajika L. Perera, Joseph D. Maman, Luca Pellegrini

**Affiliations:** Department of Biochemistry, University of Cambridge, Cambridge, United Kingdom; University of Queensland, Australia

## Abstract

**Background:**

DNA synthesis during replication relies on RNA primers synthesised by the primase, a specialised DNA-dependent RNA polymerase that can initiate nucleic acid synthesis *de novo*. In archaeal and eukaryotic organisms, the primase is a heterodimeric enzyme resulting from the constitutive association of a small (PriS) and large (PriL) subunit. The ability of the primase to initiate synthesis of an RNA primer depends on a conserved Fe-S domain at the C-terminus of PriL (PriL-CTD). However, the critical role of the PriL-CTD in the catalytic mechanism of initiation is not understood.

**Methodology/Principal Findings:**

Here we report the crystal structure of the yeast PriL-CTD at 1.55 Å resolution. The structure reveals that the PriL-CTD folds in two largely independent alpha-helical domains joined at their interface by a [4Fe-4S] cluster. The larger N-terminal domain represents the most conserved portion of the PriL-CTD, whereas the smaller C-terminal domain is largely absent in archaeal PriL. Unexpectedly, the N-terminal domain reveals a striking structural similarity with the active site region of the DNA photolyase/cryptochrome family of flavoproteins. The region of similarity includes PriL-CTD residues that are known to be essential for initiation of RNA primer synthesis by the primase.

**Conclusion/Significance:**

Our study reports the first crystallographic model of the conserved Fe-S domain of the archaeal/eukaryotic primase. The structural comparison with a cryptochrome protein bound to flavin adenine dinucleotide and single-stranded DNA provides important insight into the mechanism of RNA primer synthesis by the primase.

## Introduction

The antiparallel nature of the DNA helix and the obligate 5′ to 3′ direction of DNA polymerase activity means that synthesis must initiate at least once on the leading strand and multiple times on the lagging strand during replication [1]. Organisms in all kingdoms of life have evolved specialised polymerases, termed primases, that are endowed with the unique ability to synthesise short RNA primers from a pool of ribonucleotides [2]. During replication, the primase is a constitutive component of the replisome, the protein complex associated with the replication fork [3]. In eukaryotic and archaeal organisms, the primase is a heterodimeric enzyme consisting of a small (PriS) and a large subunit (PriL) [4]. PriL is an essential gene in yeast [5] and has a critical but poorly understood role in primase activity [6], [7], [8], [9]. A crystallographic model of an archaeal heterodimeric primase elucidated the mode of interaction of the two subunits but could not clarify the functional role of the PriL in primase function [10].

Recently, it has been reported that a conserved sequence in the C-terminal region of archaeal and eukaryotic PriL folds in a separate domain around an Fe-S cluster cofactor coordinated by four conserved cysteines (PriL-Carboxy-Terminal Domain; PriL-CTD) [11], [12]. Biochemical and genetic evidence shows that the PriL-CTD performs an essential function during initiation of RNA primer synthesis [8], [11]. In order to improve our knowledge of the mechanism of initiation of DNA synthesis in eukaryotic replication, we have determined the crystal structure of yeast PriL-CTD. The structure reveals a novel architecture for a Fe-S domain protein, rationalises several biochemical and genetic observations concerning the eukaryotic primase and provides unexpected clues about the mechanism of RNA primer synthesis in eukaryotic replication.

## Results and Discussion

### Structure of the PriL-CTD

A region spanning the Fe-S domain (residues 316 to 512) of the large primase subunit from *Saccharomyces cerevisiae* was expressed and purified in *E. coli* and its X-ray crystal structure was determined at 1.55 Å (see Material and Methods and Table 1 for X-ray diffraction data collection and refinement statistics). The last sixteen residues of PriL were excluded from our analysis because poorly conserved and predicted to be conformationally disordered.

**Table 1:** X-ray data collection and refinement statistics.

Data collection^1^	
Space group	P6_1_
a, b, c (Å)	86.6, 86.6, 141.5
Unique chains in a.s.u.	2
Wavelength (Å)	0.9793
Unique reflections	88345
Completeness (%)	100.0 (99.8)
Multiplicity	22.4 (20.7)
R_sym_(%)^2^	0.101 (0.708)
I/σ(I)	21.6 (4.4)
Refinement statistics	
Resolution	1.55
Number of unique reflections	83841
R_work_ 3	0.154
R_free_ 4	0.164
Number of non-H atoms	
Protein	3163
[4Fe-4S]	16
Waters	496
*B*-factors (Å^2^)	
All atoms	26.5
Protein	24.1
[4Fe-4S]	14.9
Water	40.6
R.m.s.d.	
Bond lengths (Å)	0.020
Bond angles (°)	2.40
Ramachandran plot	
Favoured region (%)	99.5
Allowed region (%)	100.0
Outliers (%)	0.0

1. Figures in parenthesis refer to the outermost shell.

2. 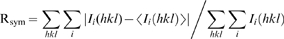

3. 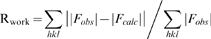

4. 5% of unique reflections were set aside for R_free_ calculations.

**Figure 1:**
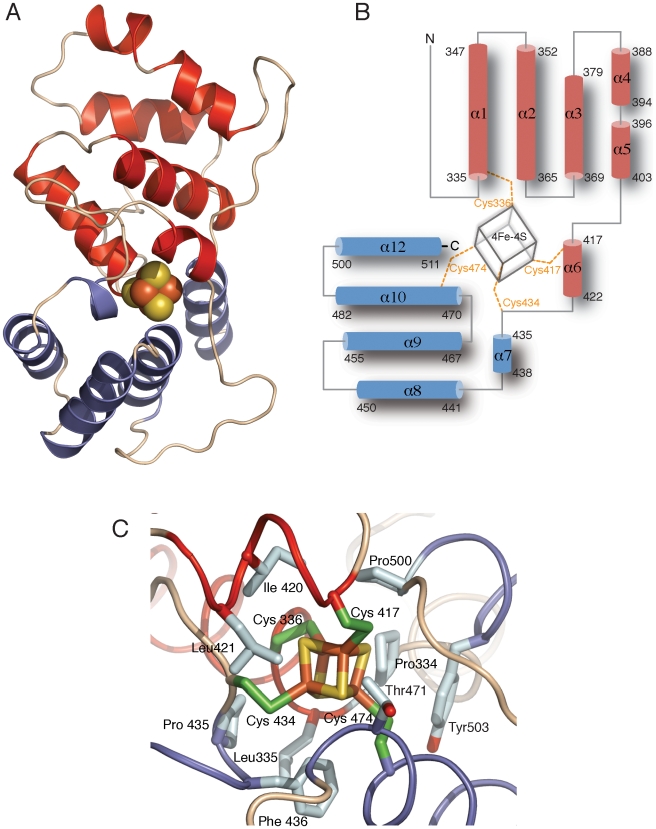
Crystal structure of yeast PriL-CTD at 1.55 Å. (**A**) Ribbon diagram of the PriL-CTD. The larger N-terminal domain is coloured in red and the smaller C-terminal domain in blue. The Fe-S cluster cofactor is shown in space-fill representation; the Fe and S atoms are in red and yellow, respectively. (**B**) Topology diagram of the PriL-CTD; the numbering and residue span of each alpha helix is indicated. The cysteine residues that ligate the Fe-S cluster are explicitly indicated. Colouring as in panel (A). (**C**) Close-up view of the Fe-S cluster. Hydrophobic residues surrounding the Fe-S cluster are shown. The main-chain trace of the PriL-CTD is shown as a thin tube, whereas the Fe-S cluster and side chains are drawn as sticks.

The PriL-CTD folds in a highly elongated shape, which results from the stacking of 11 alpha-helices in a broadly perpendicular fashion to the longer axis of the structure (**Figure 1A and 1B**). The most conspicuous feature of the PriL-CTD architecture is its organisation in two largely independent domains, arranged as triangular wedges joined via their thin ends. The larger N-terminal domain spans residues 321 to 433 whereas the smaller C-terminal domain includes residues 434 to 512. The Fe-S cluster is located at the seam that joins the two domains; each domain contributes two of the four cysteines that bind the cluster: Cys 336 and Cys 417 in the N-terminal domain and Cys 434 and 474 in the C-terminal domain (**Figure 1B**). The structure of the PriL-CTD confirms the prediction of the Fe-S cluster cofactor as a cubane-like [4Fe-4S] cluster; inspection of the electron density surrounding the cluster during refinement reveals full occupancy of the atomic positions of the cluster (**Figure S1**). Thus, the cluster appears to have been incorporated fully and stably in the recombinant protein and no Fe atom is lost during protein purification and crystallization.

Despite its inter-domain location, the Fe-S cluster is almost completely shielded from solvent by a highly hydrophobic environment, formed by the side chains of residues in both N-terminal (Pro 334, Leu 335, Ile 420, Leu 421) and C-terminal domain (Pro 435, Phe 436, Thr 471, Pro 500, Tyr 503) (**Figure 1C**). The shared coordination of the Fe-S cluster by the two domains indicates that the cluster has an important architectural role in defining their relative orientation. Thus, the crystal structure rationalises previous experimental evidence showing that the integrity of the Fe-S cluster is required for primase activity and structural stability of this domain [11]. Interestingly, the C-terminal domain is almost completely absent in the PriL-CTD of archaeal primases; structure-based sequence alignment identifies only helix 11, connected to the N-terminal domain by a variable linker region bearing the two C-terminal cysteine ligands (**Figure S2**). These observations suggest that the cluster in the archaeal primase might be partially solvent exposed, which would contribute to explain its decreased stability under aerobic conditions observed for the archaeal primase from *Sulfolobus solfataricus* [11].

### Structural similarity between PriL-CTD and DNA Photolyase

When the structure of the yeast PriL-CTD was compared to protein structures in Protein Data Bank in Dali [13] no significant structural similarity with known Fe-S proteins was detected, confirming that the PriL-CTD represents a novel type of Fe-S cluster-binding domain. Surprisingly, the search identified significant matches, with Z-scores ranging from 8.0 to 6.1 (**Table S1**), between the larger N-terminal domain of PriL-CTD and members of the DNA photolyase/cryptochrome family, DNA repair enzymes that revert the accidental cross-linking of adjacent bases, such as cyclobutane-pyrimidine dimers (CPD) and pyrimidine-pyrimidone (6-4) dimers, caused by ultraviolet light [14]. DNA photolyases and chryptochromes share a strongly conserved structure, consisting of an N-terminal α/β domain and a C-terminal α-helical domain harbouring a flavin adenine-dinucleotide (FAD) cofactor that is essential for catalysis [15]. Binding to UV-damaged DNA involves an extra-helical extrusion of the cross-linked dinucleotide into the active site of the enzyme. DNA repair takes place by a photochemical reaction involving the transfer of a photon to the substrate from an activated FAD molecule located near the active site of the enzyme [16].

**Figure 2:**
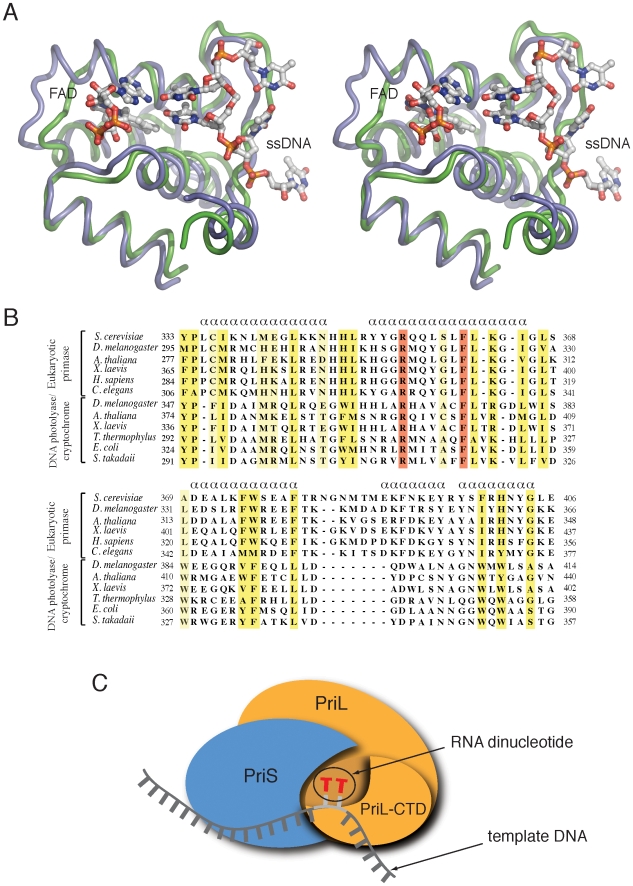
Structural similarity of the PriL-CTD and DNA photolyase. (**A**) Cross-eye stereo-diagram of the N-terminal domain of PriL-CTD superimposed on the active site region of cryptochrome Cry3 from *A. thaliana*, bound to FAD and ssDNA. The PriL-CTD is drawn as a thin tube in blue, the DNA photolyase in green. FAD and ssDNA are shown as sticks, coloured according to element type. (**B**) Structure-based alignment of a representative set of primase and photolyase sequences spanning the region of structural homology (GenBank protein ID: *S. cerevisiae*, AAA34900; *D. melanogaster*, AAG01548; *A. thaliana*, AAM61309; *X. laevis*; AAH88966; *H. sapiens*, AAH64931; *C. elegans*, CAB03469; *D. melanogaster*, BAA12067; *A. thaliana*, BAC65244; *X. laevis*, AAI69685; *T. thermophilus*, AAS82388; *E. coli*, CAR11998; S. tokodaii, BAB65903). Absolutely conserved positions are highlighted in red, strongly conserved positions in yellow and weakly conserved positions in pale yellow. The alpha helical elements in the alignment are indicated above the sequences. (**C**) Cartoon of the heterodimeric primase, showing a possible model for the essential role of the PriL-CTD in initiation of RNA primer synthesis. According to the model, the PriL-CTD would assist the catalytic subunit PriS in the simultaneous binding of the two initial ribonucleotides and in promoting their Watson-Crick base pairing at the initiation site on the template DNA.

The portion of the DNA photolyase/cryptochrome structure identified as similar to the N-terminal domain of PriL-CTD encompasses its active site and includes the FAD- and DNA-binding sites [17], [18]. The two structures show a remarkable degree of three-dimensional similarity: residues 334 to 423 (80 amino acids) in the N-terminal domain of the PriL-CTD, can be superposed on residues 373 to 457 (85 amino acids) of DASH cryptochrome 3 from *A. thaliana* (PDB id: 2VTB) with an Cα rmsd of 1.75 Å (**Figure 2A**). The structural conservation applies not only to the secondary structure elements but includes also the intervening loops. A structure-based alignment of primase and photolyase/cryptochrome sequences over the structurally homologous region reveals that their common architecture is underpinned by the conservation of several hydrophobic and aromatic residues, such as Pro 334, Leu 344, Phe 361, Leu 362, Ile 365, Leu 367, Phe 374, Trp 375 and His 401 (**Figure 2B**).

The structural similarity with the active site of the DNA photolyase/cryptochrome class of enzymes provides potential insight into the critical yet poorly understood role of the PriL-CTD in primase function [11], [12]. The mode of binding of single-stranded (ss) DNA and FAD observed in the co-crystal structure of the DASH cryptochrome 3 from *A. thaliana* suggests that the PriL-CTD could adopt a similar mode of interaction with the template DNA and ribonucleotides during *de novo* RNA synthesis. In particular, the spacial relationship between FAD and the extruded, cross-linked pyrimidine dimer observed in the active site of DNA photolyase suggests a possible arrangement for the pairing of the first dinucleotide of the RNA primer onto template DNA during the initiation step catalysed by the primase. Thus, our crystallographic analysis suggests a structural rationale for how the PriL-CTD would participate in RNA primer synthesis: by assisting the catalytic subunit PriS in the simultaneous binding of the two initial RNA nucleotides and concomitantly by promoting dinucleotide base-pairing with template DNA at the initiation site (**Figure 2C**).

**Figure 3:**
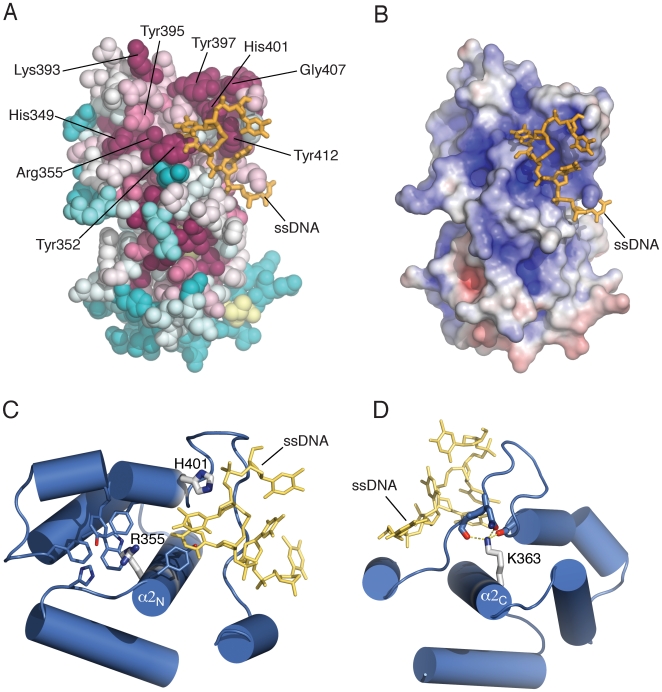
Structural features of the yeast PriL-CTD. (**A**) Amino acid conservation mapped on the crystal structure of the PriL-CTD. The evolutionary conservation analysis of surface residues was performed with the ConSurf server [28], based on 49 sequences of eukaryotic primases. Degree of conservation is shown by colour range, from magenta (highest conservation) to cyan (lowest). The structure is shown in space fill representation. Highly conserved residues that might be important for the functional role of the PriL-CTD are indicated. The ssDNA molecule of the DASH cryptochrome 3 co-crystal (PDB id: 2VTB), superimposed on the PriL-CTD, is shown in stick representation. (**B**) Electrostatic potential of the PriL-CTD, mapped on its solvent-accessible surface at contouring levels of ±5 kT. Positive charge is in blue, negative charge in red. The potential was calculated using APBS [29] in PyMol (http://www.pymol.org/). The ssDNA is depicted as in panel B. (**C**) and (**D**) Structural details of PriL-CTD residues known to have important functional roles in primase function. Panel C depicts Arg 355 and His 401, panel D shows Lys 363. Please see text for the functional interpretation of their role. The side chains are shown as thick or thin sticks, the helices as cylinders. The ssDNA from the cryptochrome 3 co-crystal structure is also shown in yellow. Polar interactions involving Lys 363 are drawn as dashed yellow lines.

Strong supporting evidence in favour of this hypothesis is provided by the following observations. Inspection of evolutionary conservation of surface residues in the PriL-CTD reveals that a majority of conserved residues are located in the region of the PriL-CTD that is homologous to the DNA photolyase (**Figure 3A**). In particular, basic residues at positions 355 and 363 of the yeast PriL-CTD, which are strictly conserved among primases and photolyases, have critical functional roles in RNA primer synthesis [8], [11]. The crystal structure of the yeast PriL-CTD shows that Arg 355 on helix 2 is surrounded by aromatic residues that keep it poised for potential interaction with both RNA nucleotides and template DNA (**Figure 3C**). Lys 363 on helix 2 plays an important architectural role via multiple polar interactions with main-chain carbonyl moieties of residues Asn 402 and Tyr 412 and side-chain amide function of Asn 411, thus fixing the conformation of the putative DNA-binding loop linking helices 5 and 6 (**Figure 3D**). Missense mutation of His 401, which occupies a highly conserved aromatic residue position in primases and photolyases, is lethal in yeast, suggesting that it has an essential functional role [19]. The PriL-CTD structure shows that the side chain of His 401 on helix 4 would be ideally located to interact with template DNA (**Figure 3C**).

Furthermore, analysis of the electrostatic potential on the surface of the PriL-CTD reveals a region of positive electrostatic potential in the region of homology with DNA photolyase (**Figure 3B**). This observation is consistent with the presence of highly conserved basic and polar residues (His 349, Arg 351, Lys 390, Lys 393 and His 401) that could interact with the phosphate backbone of template DNA and the substrate ribonucleotides. In addition, the putative active site region of the PriL-CTD includes several conserved aromatic residues, Tyr 352, Tyr 395, Tyr 397 and Tyr 412 that are solvent exposed and could provide points of contact through aromatic stacking with the bases of the DNA template.

### DNA binding properties of the PriL-CTD

**Figure 4:**
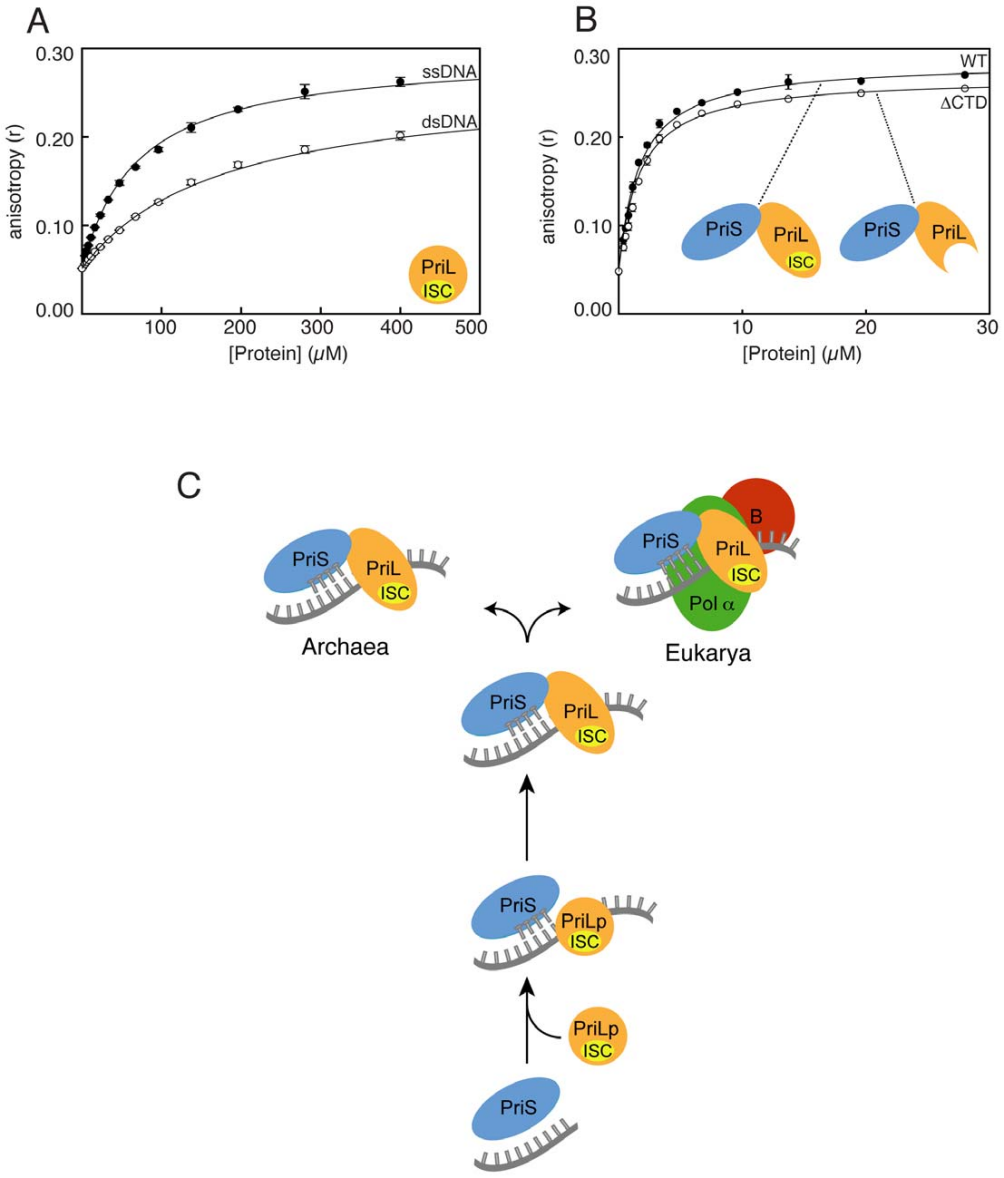
The PriL-CTD binds DNA. (**A**) Fluorescence anisotropy curves of fluorescein-labelled DNA in the presence of increasing amounts of PriL-CTD. Each data point is the average of three independent measurements. (**B**) Fluorescence anisotropy curves of fluorescein-labelled DNA in the presence of full-length (WT) or truncated primase, missing the PriL-CTD (ΔCTD). Each data point is the average of three independent measurements. (**C**) Cartoon of the possible evolutionary history of the PriL. The PriL might have derived from a smaller Fe-S protein (PriLp: PriL precursor; ISC: Iron-Sulfur Cluster) with moderate affinity for DNA, reflecting an unknown function in nucleic acid metabolism. Recruitment of PriLp to a ‘prim’-fold polymerase, the ancestor of current PriS, would have allowed initiation of DNA synthesis during replication. Over time, the PriLp would have become incorporated in a constitutive heterodimer with PriS.

The structural similarity of the N-terminal domain of PriL-CTD with the active site of DNA photolyase and the analysis of its electrostatic potential and surface-residue conservation predicts that the PriL-CTD should possess a DNA binding ability. In order to investigate the DNA-binding properties of the PriL-CTD, we assessed its ability to bind to fluorescein-labelled DNA by fluorescence anisotropy. Fluorescence anisotropy measurements of ssDNA 20mer in the presence of increasing amounts of PriL-CTD produced an equilibrium dissociation constant (K_D_) of 70±0.2 µM (**Figure 4A**). The binding of the PriL-CTD to ssDNA was independent of the sequence content as a similar K_D_ was measured using a poly(dT) ssDNA of the same size (data not shown). In contrast, the affinity of the PriL-CTD for double-stranded (ds) DNA was approximately 2.5 weaker, at 178±1.3 µM (**Figure 4A**). Similar values for the affinity of the PriL-CTD towards ss- and dsDNA was observed by surface plasmon resonance with biotin-labelled DNA immobilised on a streptavidin-chip (**Figure S3**).

The rather low DNA-binding affinity of the PriL-CTD agrees with previous biochemical evidence that the DNA-binding ability of the eukaryotic primase is mainly provided by the small subunit PriS [9]. Using fluorescence anisotropy, we determined that the full-length heterodimeric yeast primase binds a poly(dT) DNA 40mer with a K_D_ of 1.7 µM and that deletion of the PriL-CTD does not impair affinity (**Figure 4B**). These observations suggest that the PriL-CTD is not essential for binding to template DNA but relies on the rest of the heterodimeric primase for its recruitment to the initiation site. Instead, interaction with template DNA must be important for the correct positioning of the PriL-CTD relative to the active site of the small subunit during the initiation step.

The detection of micromolar affinity for nucleic acid is consistent with our proposed mechanism that involves binding to ssDNA as part of the essential function of PriL-CTD in RNA primer synthesis. Thus, several pieces of evidence, such as the strong sequence conservation of the PriL-CTD in archaeal and eukaryotic primases, its ability to fold autonomously and its affinity to DNA concur to suggest that the precursor of the current PriL-CTD was an ancient Fe-S protein that was recruited to DNA replication at an early stage of cellular evolution (**Figure 4C**).

The observation of a strong structural similarity with the active site region of DNA photolyase raises the intriguing possibility that the role of the PriL-CTD in primase activity derives from an ancestral DNA repair function associated with recognition of cyclobutane-pyrimidine dimers, a very common type of DNA lesion. We speculate that the Fe-S cluster would have been a suitable cofactor to provide the reduction potential for the repair of the CPD crosslink. A vestige of this origin might survive today in the strong preference of eukaryotic primases for initiating on poly-pyrimidine stretches of template DNA[20].

## Materials and Methods

### Cloning, expression and purification

Residues 316 to 512 of *S. cerevisiae* DNA primase large subunit were cloned into an RSF1-Duet expression vector (Novagen) in fused to an N-terminal six-histidine tag. We observed during purification of recombinant PriL-CTD that the loop between helices 3 and 4 was being proteolysed. We therefore engineered a PriL-CTD construct where the 381-RNG-383 sequence was excised. The protein was expressed by IPTG induction in *E. coli* strain BL21(DE3)Rosetta2 grown in Turbo broth (Molecular Dimensions Ltd) at 20°C and purified by Co-NTA and heparin chromatography, followed by TEV protease cleavage of the tag and size-exclusion chromatography. The purified protein was concentrated to 7 mg/ml in 20 mM Hepes pH 6.8, 200 mM KCl and 3 mM TCEP, flash frozen in liquid nitrogen and stored at −80°C. The *S. cerevisiae* full-length and C-terminally truncated (ΔCTD) primase were expressed and purified as described previously [11].

### Crystallization, phasing and data refinement

Seleno-methionine (Se-Met) incorporation for anomalous diffraction phasing was achieved by the metabolic inhibition procedure as described [21]. Se-Met labelled PriL-CTD was crystallized by vapour diffusion at 18°C, mixing 2 µl of protein solution (7 mg/ml) with 2 µl of crystallization buffer containing 100 mM TrisCl, pH 7.5, 1 mM zinc acetate and 11% ethanol. The crystal structure of the PriL-CTD was determined using phase information derived from anomalous scattering data collected at the Se K-edge at beamline PXIII of the Swiss Light Source (SLS) in Zurich, Switzerland. The crystals belong to the space group P6_1_ (a = b = 86.6 Å, c = 141.5 Å) with two copies of the protein in the asymmetric unit. An initial model of the PriL-CTD was obtained using PHENIX [22], completed manually in Coot [23] and refined in REFMAC5 [24] and Buster [25] to R and R_free_ values of 0.154 and 0.164, respectively at 1.55 Å resolution. Residues 512, 483 to 494 in chain A and 316 in chain B were not visible in the electron density map; therefore they were considered to be disordered and were not included in the crystallographic model. 99.5% of residues were in the favoured regions of the Ramachandran plot with no outliers. The MolProbity [26] score for the refined model is 1.12, in the 99^th^ percentile of structures refined at comparable resolution.

### DNA binding assays

Biotin and fluorescein 5′-labeled DNA oligonucleotides were purchased from Sigma (5′ biotin-ACTTCCATTCTCCTCTCACC-3′; 5′ fluorescein-GGTGAGAGGAGAATGGAAGT-3′). The sequence was designed using the NUPACK software package (http://www.nupack.org/) in order to minimize unwanted secondary structure formation. A labeled double-strand DNA (dsDNA) oligo was prepared by annealing the two complementary oligos by cooling from 95°C to 25°C at 1°C/minute in a PCR thermocycler.

Fluorescence anisotropy measurements were recorded in a PHERAstar Plus multi-detection plate reader (BMG Labtech) equipped with fluorescence polarization optic module (λ_ex_  = 485 nm; λ_em_  = 520 nm), at 25°C. Each data point is the mean of 200 flashes per well. The voltage gain was set by adjusting the target mP values of fluorescein-labeled oligos relative to that of fluoresceine (35 mP). Serial dilutions of proteins (in triplicates) were made in buffer F (20 mM HEPES pH 6.8, 150 mM KCl, 1 mM EDTA), and 20 nM (wild-type primase and ΔCTD-primase) or 80 nM (PriL-CTD) of fluorescein-labeled DNA oligos. Protein concentrations were determined spectroscopically using λ_280_ extinction coefficient of 36,900 M^−1^cm^−1^ for PriL-CTD and 122,130 M^−1^cm^−1^ and 81,875 M^−1^cm^−1^ for WT-Primase and ΔCTD-primase, respectively. The results were analysed assuming a one-to-one binding model as previously described [27].

SPR measurements were performed in a Biacore T100 (GE Healthcare) at 25°C and 30 µl/min using buffer F containing 0.005% P20 (Polyoxyethylene (20) sorbitan monolaurate) and no DNA. Biotinylated ssDNA and dsDNA oligos were attached to flow channel 2 and 4 (FC2 and FC4 respectively) of a streptavidin-coated sensor chip. The DNA binding levels were 81 RU (response units) and 167 RU for ssDNA and dsDNA respectively. PriL-CTD protein in buffer F containing 0.005% P20 was injected at several concentrations for 60 seconds and allowed to dissociate for 60 seconds. The surface was regenerated with 2×60 seconds injections of 1 M NaCl. No loss of signal due to oligo dissociation was detected during the experiments. The data was processed by subtracting background binding to the reference flow channels (FC1 & FC3). The corrected sensograms were analysed using a steady state affinity one-to-one model with bulk refractive index contribution factor (Biacore, GE HealthCare).

## Supporting Information

Figure S1 Omit map of the Fe-S cluster. The Fo-Fc electron density map (coloured in green) was calculated by omitting the Fe-S cluster in the late stages of refinement and is contoured at 12 sigma. The Fe-S cluster and the side-chains of the four cysteine ligands are drawn as sticks. Colouring as in Figure 1 of the main text.
(0.51 MB PDF)

Figure S2 Structure-based alignment of eukaryotic and archaeal PriL-CTD sequences. Completely conserved residues are highlighted in green, conserved residues in cyan and identical residues in at least 50% of sequences in yellow. Coloured arrows (red for the N-terminal domain and blue for the C-terminal domain) above the alignment indicated the position and extent of alpha helices in the PriL-CTD structure.
(0.68 MB PDF)

Figure S3 Surface plasmon resonance analysis of the PriL-CTD interaction with ssDNA (A) and dsDNA (B). Several runs covering a range of PriL-CTD concentrations from 0 to 500 µM are shown. The insets show the fit of the RU values at equilibrium (Req) plotted against the PriL-CTD concentration.
(0.33 MB PDF)

Table S1 Dali search of the PriL-CTD structure against the Protein Data Bank. The table reports the top seven hits resulting from the Dali search. Chain B was used in the search as it contains an uninterrupted polypeptide chain. Comparable results were obtained for chain A.
(0.04 MB DOC)
