# Supplementary figures and images for: Shared Active Site Architecture between the Large Subunit of Eukaryotic Primase and DNA Photolyase
Source: PLoS One. 2010 Apr 9;5(4):e10083. doi: 10.1371/journal.pone.0010083 (PMC2852410; doi:10.1371/journal.pone.0010083)

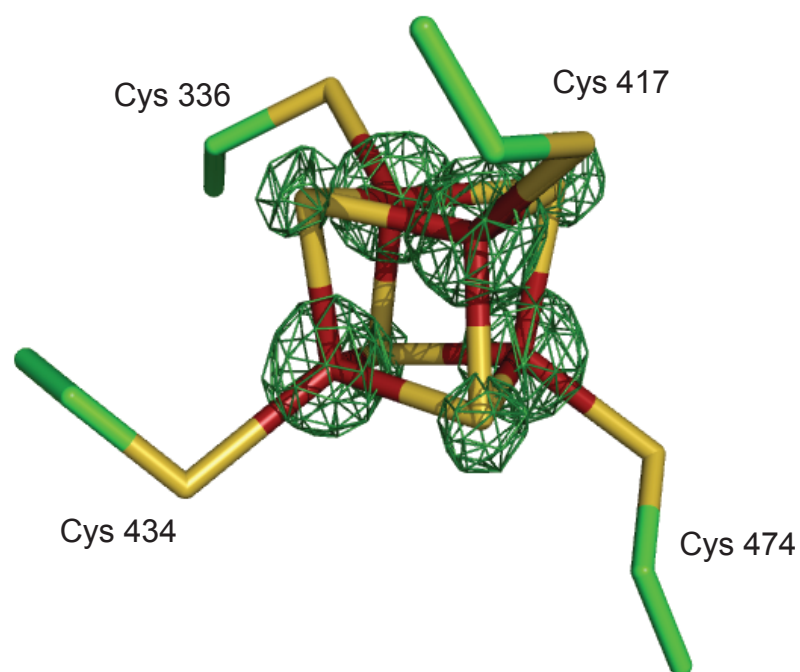

Figure S1

Supplement: Figure S1 — Omit map of the Fe-S cluster. The Fo-Fc electron density map (coloured in green) was calculated by omitting the Fe-S cluster in the late stages of refinement and is contoured at 12 sigma. The Fe-S cluster and the side-chains of the four cysteine ligands are drawn as sticks. Colouring as in Figure 1 of the main text. (0.51 MB PDF) [file pone.0010083.s001.pdf]

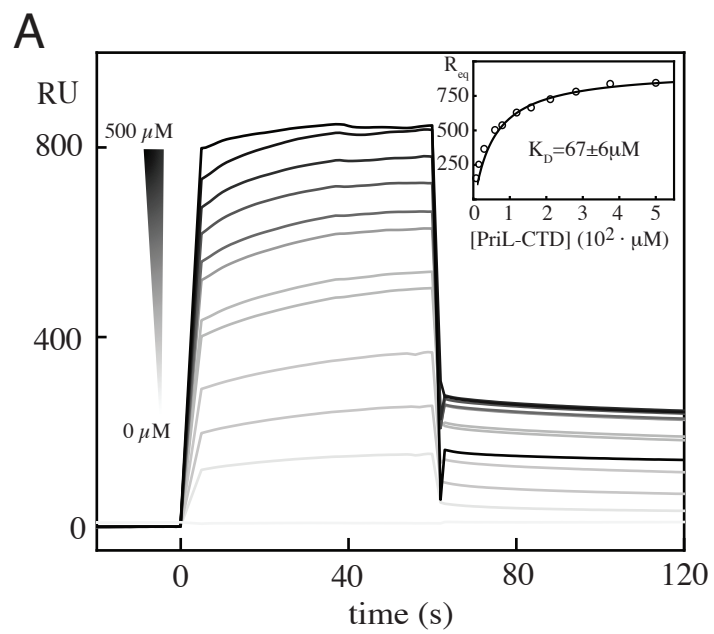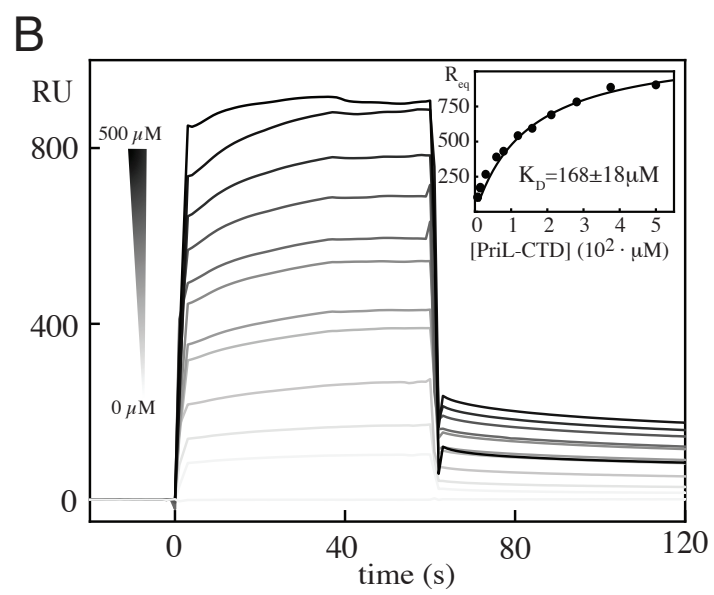

Figure S3

Supplement: Figure S3 — Surface plasmon resonance analysis of the PriL-CTD interaction with ssDNA (A) and dsDNA (B). Several runs covering a range of PriL-CTD concentrations from 0 to 500 µM are shown. The insets show the fit of the RU values at equilibrium (Req) plotted against the PriL-CTD concentration. (0.33 MB PDF) [file pone.0010083.s003.pdf]
