# Supplementary material for: Shared Active Site Architecture between the Large Subunit of Eukaryotic Primase and DNA Photolyase
Source: PLoS One. 2010 Apr 9;5(4):e10083. doi: 10.1371/journal.pone.0010083 (PMC2852410; doi:10.1371/journal.pone.0010083)
Supplement: Table S1 — Dali search of the PriL-CTD structure against the Protein Data Bank. The table reports the top seven hits resulting from the Dali search. Chain B was used in the search as it contains an uninterrupted polypeptide chain. Comparable results were obtained for chain A. (0.04 MB DOC) [file pone.0010083.s004.doc]

| **PDB id** | **Z** | **rmsd** | **lali** | **nres** | **%id** | **Description** |
| --- | --- | --- | --- | --- | --- | --- |
| **2vtb-A** | 8.0 | 2.9 | 103 | 501 | 13 | CRYPTOCHROME DASH |
| **2j07-A** | 7.3 | 3.3 | 103 | 419 | 8 | DEOXYRIBODIPYRIMIDINE PHOTO-LYASE |
| **1qnf** | 7.2 | 2.8 | 101 | 475 | 6 | PHOTOLYASE |
| **1dpn-A** | 7.1 | 2.9 | 101 | 470 | 6 | DNA PHOTOLYASE |
| **2e0i-B** | 7.0 | 2.7 | 98 | 431 | 6 | 432AA LONG HYPOTHETICAL DEOXYRIBODIPYRIMIDINE |
| **3fy4-A** | 6.1 | 3.1 | 101 | 522 | 6 | 6-4 PHOTOLYASE |
| **3cvv-A** | 6.1 | 3.1 | 100 | 519 | 8 | RE11660P |

**Supplementary Table 1.** Top seven hits of the Dali search of the PriL-CTD structure against the Protein Data Bank. Chain B was used in the search as it contains an uninterrupted polypeptide chain. Comparable results were obtained with chain A.
