# Supplementary material for: Shared Active Site Architecture between the Large Subunit of Eukaryotic Primase and DNA Photolyase
Source: PLoS One. 2010 Apr 9;5(4):e10083. doi: 10.1371/journal.pone.0010083 (PMC2852410; doi:10.1371/journal.pone.0010083)
Supplement: Figure S2 — Structure-based alignment of eukaryotic and archaeal PriL-CTD sequences. Completely conserved residues are highlighted in green, conserved residues in cyan and identical residues in at least 50% of sequences in yellow. Coloured arrows (red for the N-terminal domain and blue for the C-terminal domain) above the alignment indicated the position and extent of alpha helices in the PriL-CTD structure. (0.68 MB PDF) [file pone.0010083.s002.pdf]

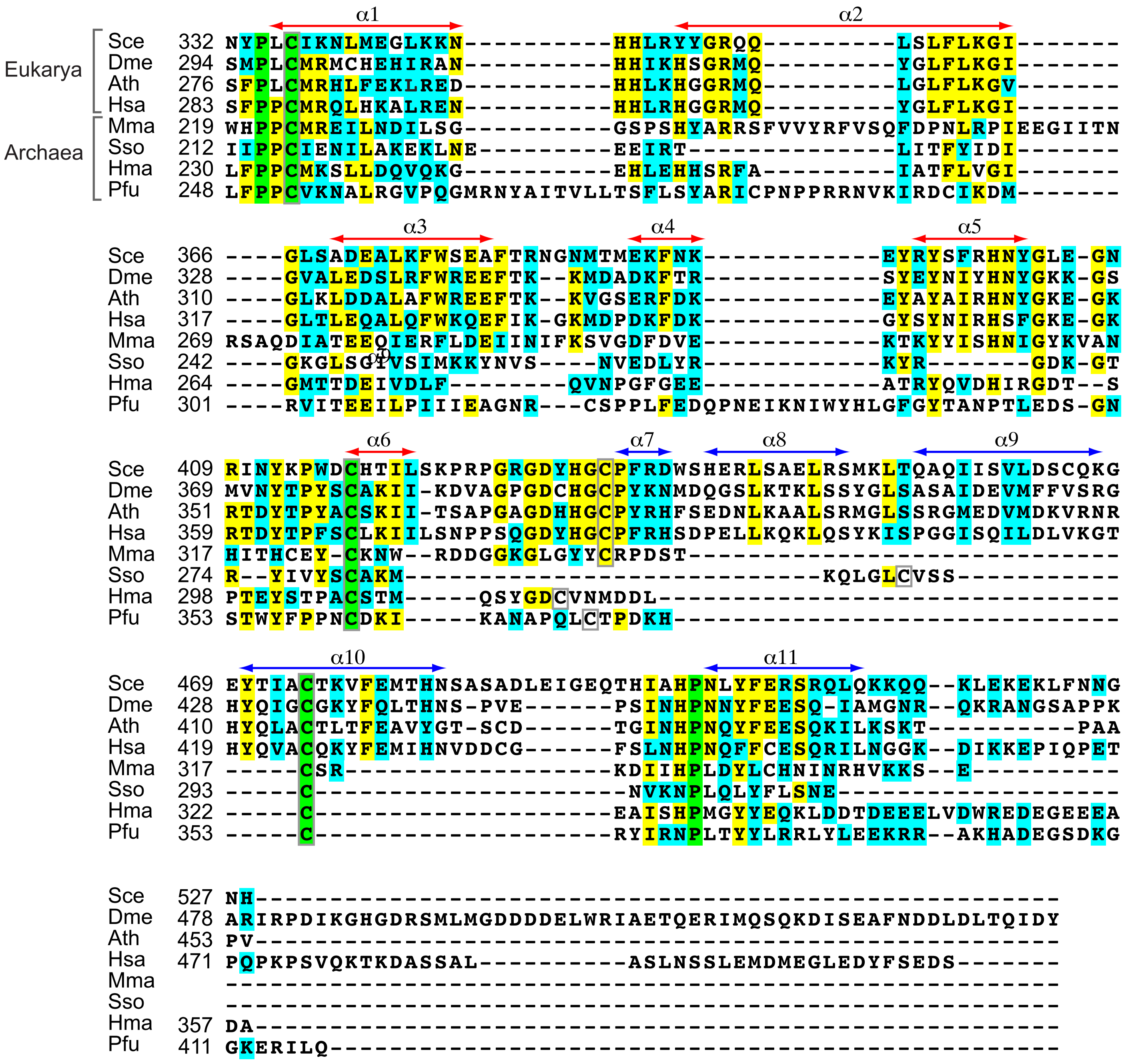
α1
α2
α3
α4
α5
α6
α7
α8
α9
α10
α11
Eukarya
Archaea
Sce
Dme
Ath
Hsa
Mma
Sso
Hma
Pfu

Figure S2
